# Preliminary Feasibility and Dosimetric Evaluation of Retroperitoneal Hydrogel Spacer Placement for Stereotactic Body Radiation Therapy: A Cadaveric Study

**DOI:** 10.1016/j.adro.2026.102211

**Published:** 2026-09-12

**Authors:** Shin Tanabe, Kotaro Yoshio, Takaaki Komiyama, Soichi Sugiyama, Toshiya Kameoka, Ryota Kimura, Takahiro Kawabata, Yuta Otsuka, Hideki Aoyama, Kenta Watanabe, Ayano Kawaguchi, Takao Hiraki

**Affiliations:** a Department of Proton Beam Therapy, Faculty of Medicine, Dentistry and Pharmaceutical Sciences, Okayama University, Okayama, Japan; b Department of Radiology, Graduate School of Medicine, Dentistry and Pharmaceutical Sciences, Okayama University, Okayama, Japan; c Department of Human Morphology, Graduate School of Medicine, Dentistry and Pharmaceutical Sciences, Okayama University, Okayama, Japan; d Department of Radiology, Tsuyama Chuo Hospital, Okayama, Japan; e Division of Radiological Technology, Okayama University Hospital, Okayama, Japan; f Department of Radiology, Medical Development Field, Okayama University, Okayama, Japan; g Department of Radiology, Faculty of Medicine, Dentistry and Pharmaceutical Sciences, Okayama University, Okayama, Japan

## Abstract

**Purpose:**

Hydrogel spacers reduce the rectal radiation dose and toxicity in prostate cancer radiation therapy. However, their use in other malignancies, including renal cell carcinoma (RCC), remains underexplored. This study aimed to explore the feasibility of hydrogel spacer placement at different retroperitoneal sites and its potential dosimetric effects during stereotactic body radiation therapy for RCC using cadaveric models.

**Methods and Materials:**

Hydrogel spacers were percutaneously administered under computed tomography (CT) guidance to 3 cadavers at the following retroperitoneal sites: (1) the perirenal space, (2) the perivascular region of the iliac vessels, and (3) the iliacus muscle region. Spacer stability and separation distance from organs at risk were evaluated using CT images acquired immediately after placement, at 1 week, and at 1 to 2 months. The dosimetric effect was assessed in the perirenal spacer case by comparing radiation treatment plans for a simulated RCC lesion generated before and after spacer placement using a treatment planning system.

**Results:**

Hydrogel spacer placement in the perirenal space achieved a stable separation of approximately 1 cm between the kidney and bowel, which was maintained at the 1-month follow-up. The perivascular spacer failed to maintain its shape at the time of placement and became indistinguishable on the follow-up CT, whereas the iliacus muscle spacer remained stable but provided limited bowel separation. Treatment planning showed a reduction in bowel dose in the perirenal case, with the maximum dose to the small intestine reduced by 9.6% and to the large intestine by 35.9%.

**Conclusions:**

This exploratory cadaveric study suggests that CT-guided hydrogel spacer placement in the perirenal space may be technically feasible and may reduce the bowel dose in simulated RCC stereotactic body radiation therapy. However, the other retroperitoneal locations evaluated in this study showed less favorable spacer behavior or limited bowel separation. Further studies are required to validate these preliminary findings.

## Introduction

In radiation therapy, the proximity between the tumor and bowel can lead to excessive radiation exposure, increasing the risk of complications such as ulcers, bleeding, and perforation.^1^ Creating physical separation between the tumor and bowel may reduce the bowel dose and lower the risk of radiation-induced toxicity.

Hydrogel spacers, such as SpaceOAR (Boston Scientific), are absorbable materials that create temporary physical separation between organs at risk (OARs) and target lesions. In prostate cancer radiation therapy, hydrogel spacers have consistently been shown to reduce the rectal radiation dose and toxicity.^2^, ^3^, ^4^ These favorable outcomes have prompted interest in extending spacer applications to other malignancies, including renal cell carcinoma (RCC).

Although surgical resection remains the standard treatment for RCC, stereotactic body radiation therapy (SBRT) has emerged as an effective alternative in nonsurgical candidates. An individual patient data meta-analysis from the International Radiosurgery Consortium of the Kidney (IROCK) demonstrated excellent long-term outcomes, with a 5-year local failure rate of only 5.5% and a median estimated glomerular filtration rate decline of 14.2 mL/min/1.73 m².^5^ A major concern with SBRT for RCC is gastrointestinal toxicity because of the anatomic proximity of the kidneys to the bowel. Early dose-escalation studies reported rare but severe gastrointestinal events, such as grade 4 duodenal ulceration and gastritis.^6^^,^^7^ However, the IROCK meta-analysis reported no grade 3 toxicities and only 1 grade 4 gastrointestinal toxicity (1%) over a 5-year follow-up period.^5^ Similarly, a systematic review reported a 3% pooled incidence of treatment-related grade ≥3 adverse events,^8^ whereas the Trans-Tasman Radiation Oncology Group (TROG) 15.03 FASTRACK II trial reported approximately 10%.^9^ Despite some variability, the overall incidence of severe bowel toxicity was low with modern planning techniques and dose constraints. This favorable toxicity profile likely reflects careful patient selection (avoiding bowel-abutting tumors), strict bowel constraints, and modern image guided planning and delivery techniques.

However, tumors abutting the bowel remain a major challenge in SBRT. According to the 2025 National Comprehensive Cancer Network Guidelines for Kidney Cancer, tumors abutting the bowel should not be considered amenable to SBRT.^10^ However, methods that create sufficient separation between the tumor and bowel, such as emerging spacer technologies, may help expand the applicability of SBRT in selected cases. The clinical utility of these spacers depends on achieving a stable and sufficient separation within the retroperitoneum, which has not yet been thoroughly evaluated. Therefore, we aimed to evaluate the feasibility of computed tomography (CT)-guided placement of hydrogel spacers at different retroperitoneal sites using cadaveric models and to explore its potential dosimetric impact on the perirenal space.

## Methods and Materials

### Study design and setting

This cadaveric study was conducted at Okayama University. All donors provided written informed consent for body donation for medical education and research. The study protocol was reviewed and approved by our institutional ethics committee (approval number: K2309-026).

### Cadaver model and hydrogel spacer placement

Three Thiel-embalmed cadavers with preserved soft tissue pliability and near-life-like anatomic structures^11^^,^^12^ were used in this study. The demographic characteristics of the cadavers are summarized in Table 1. A hydrogel spacer (SpaceOAR) was administered percutaneously under CT guidance using an 18-gauge needle from the spacer kit. A total of 10 mL of hydrogel was injected into 1 assigned retroperitoneal site per cadaver (the perirenal space, perivascular region of the iliac vessels, or iliacus muscle region), thereby evaluating 3 retroperitoneal sites across the 3 cadavers. These sites were selected to represent distinct retroperitoneal anatomic environments with various degrees of fascial confinement. Although the iliacus muscle region is not typically considered a clinically relevant target for bowel displacement during SBRT, it was included as a relatively confined muscular compartment to provide an anatomic comparison with the perirenal and perivascular regions. The injection endpoint was to create approximately 1 cm of separation between the target organ and adjacent bowel, similar to the standard technique used for prostate–rectum spacer placement. Additionally, to evaluate hydrogel spacer behavior in its originally intended anatomic location, each cadaver underwent hydrogel spacer placement at the prostate–rectum interface under transrectal ultrasound guidance. Thus, each cadaver received spacers at 2 distinct locations: the prostate–rectum interface and one of the above retroperitoneal sites.

**Table 1:** Characteristics of the cadavers

Cadaver	Spacer site	Sex	Age (y)	Height (cm)	Weight (kg)	Cause of death
Cadaver 1	Perirenal space	Male	83	169	40.4	Aspiration pneumonia
Cadaver 2	Perivascular region	Male	94	158	40.8	Aspiration pneumonia
Cadaver 3	Iliacus muscle region	Male	69	165	51.0	Tongue cancer

To enhance CT visibility, 0.4 mL of Gastrografin (diatrizoate meglumine and diatrizoate sodium; Bayer) was mixed with 9.6 mL of hydrogel solution before injection.

### Imaging and stability assessment

CT was performed immediately before placement, immediately after placement, at 1 week, and at 1 to 2 months. Spacer morphology and positional stability were qualitatively assessed using serial CT images.

### Radiation treatment planning

Radiation treatment planning was performed only for the cadaver in which stable spacer placement was achieved in the perirenal space. In this cadaver, an RCC lesion with a diameter of 2 cm was simulated in the anterior part of the kidney, at the level of the renal hilum. Radiation treatment plans were generated using the Eclipse Treatment Planning System (version 16.1.2; Varian Medical Systems). Dose calculations were performed using the Acuros XB algorithm, with a dose grid resolution of 2 mm. Treatment planning was performed using a TrueBeam linear accelerator with 10 MV flattening-filter-free x-rays. SBRT was planned as a single fraction of 26 Gy using volumetric modulated arc therapy (VMAT). The clinical target volume was defined as the RCC, and the planning target volume (PTV) was created by adding a 5-mm isotropic margin to the clinical target volume. The plan was normalized such that the D99 of the PTV (the minimum dose received by 99% of the PTV) corresponded to 100% of the prescribed dose. The effect of the hydrogel spacer on the bowel dose was assessed by comparing the radiation treatment plans generated from preplacement and follow-up CT images obtained 1 month after spacer placement.

Our radiation treatment planning approach was guided by the TROG 15.03 FASTRACK II trial protocol, which served as a reference for fractionation schedules, dose prescription methods, and dose constraints for OARs in SBRT for primary RCC.^9^ However, various fractionation schemes have been reported in the literature, and the current National Comprehensive Cancer Network guidelines recognize multiple dose-fractionation approaches in SBRT for RCC.^10^ Although optimizing the VMAT plan is important, our primary goal was to ensure a fair comparison of the pre- and post-spacer plans. To achieve this, the key planning parameters, including the prescribed dose, isocenter positioning (center of the PTV), and the number and rotation angles of the VMAT arcs (2 half-rotations), were kept identical. Dose normalization was applied such that D99 of the PTV corresponded to 100% of the prescribed dose in both plans. This approach aligns with that of a previous study that aimed to minimize bias in dosimetric comparisons.^13^

## Results

### Spacer stability and separation distance

Table 2 summarizes the changes in the spacer volume, separation distance, and volume retention ratio. The final follow-up CT was performed at 1 month for the perirenal case and at 1.5 months for the iliacus case. In the perirenal space, the hydrogel spacer maintained approximately 1 cm of separation with an 87.3% volume retention rate at 1 month. Serial CT revealed minimal morphologic changes and no appreciable positional shifts during the follow-up period (Fig. 1). In contrast, the hydrogel in the perivascular region failed to maintain a discrete configuration immediately after placement and became indistinguishable on follow-up CT, precluding quantitative assessment of the spacer volume or separation distance. The iliacus muscle region exhibited good stability comparable to that of the perirenal space, although additional separation from the bowel was limited. The spacer volume in the iliacus muscle region increased 1 week after placement (Fig. 2).

**Table 2:** Hydrogel spacer stability over time

Location	Time after placement	Spacer volume (cm³)	Separation distance (cm)	Volume retention ratio (%)
Perirenal space	Immediately	11.0	1.0	100.0
	1 wk	10.3	1.0	93.6
	1 mo	9.6	0.9	87.3
Perivascular region	Immediately	NM	NM	NM
	1 wk	NM	NM	NM
	1 mo	NM	NM	NM
Iliacus muscle region	Immediately	11.5	1.0	100.0
	1 wk	14.6	1.2	127.0
	1.5 mo	10.8	1.0	93.9

*Abbreviation:* NM = not measurable.

All numerical values are reported to 1 decimal place for consistency. Computed tomography-based spacer volume measurements may have been influenced by diffusion of the Gastrografin component; therefore, calculated volume retention ratios should be interpreted with caution.

**Figure 1:**
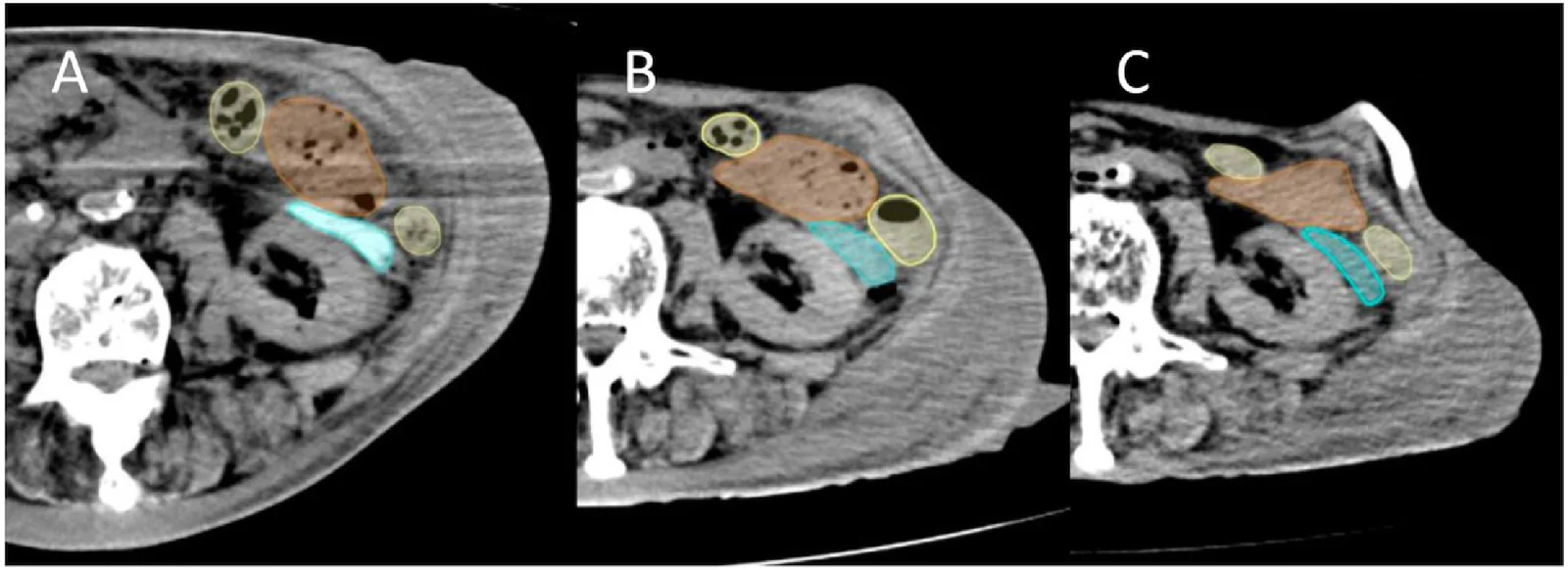
Axial computed tomography images of hydrogel spacer placement in the perirenal space. The hydrogel spacer, small intestine, and large intestine are contoured and displayed with translucent fills in cyan, orange, and yellow, respectively. The spacer is successfully positioned in the perirenal space immediately after placement (A). A stable separation of approximately 1 cm between the kidney and adjacent bowel was maintained at 1 week (B) and 1 month (C) after placement, with minimal morphologic changes observed during follow-up.

**Figure 2:**
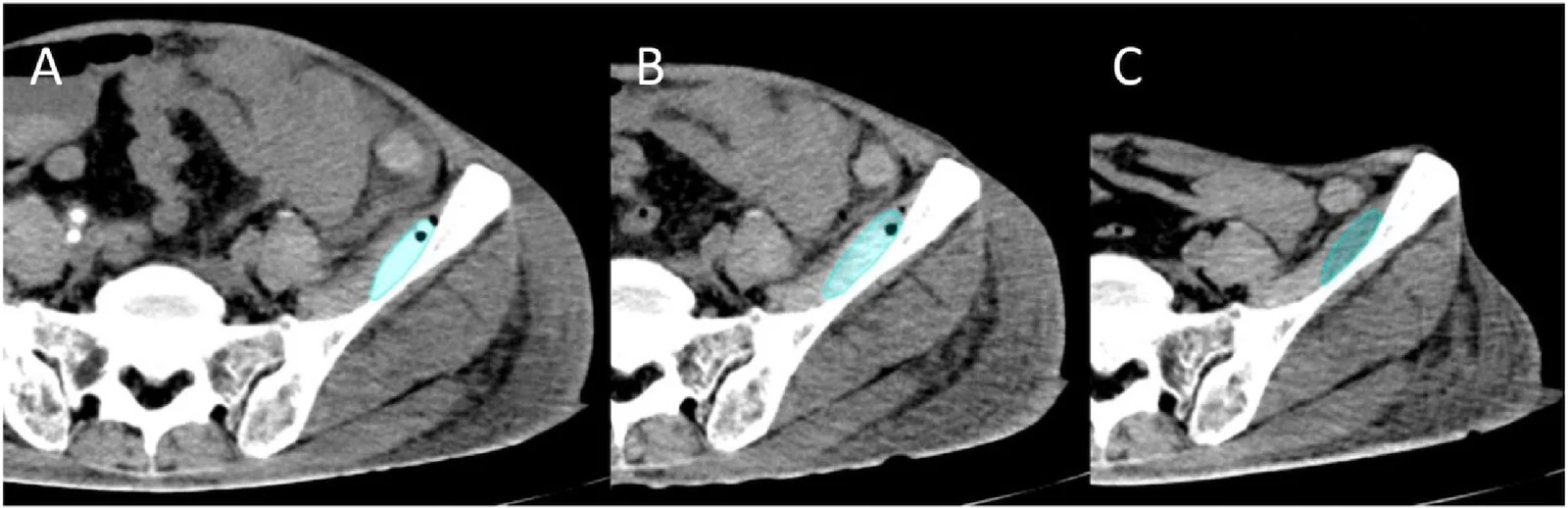
Axial computed tomography images of hydrogel spacer placement in the iliacus muscle region. The hydrogel spacer is contoured and displayed with a translucent fill in cyan. The spacer is successfully placed in the iliacus muscle region immediately after placement (A). An apparent increase in spacer volume is observed 1 week after placement (B). The spacer remains identifiable 1.5 months after placement (C).

In addition to retroperitoneal placement, the hydrogel spacer was evaluated at the prostate–rectum interface to verify its behavior at its originally intended anatomic site. In 1 cadaver, the spacer maintained a stable separation of 1 cm at 1 week and 1 month postplacement. However, in another cadaver, imaging artifacts from a prior total hip replacement made evaluation difficult, and in the third, a large volume of ascites impaired assessment of spacer morphology and position.

### Radiation dose reduction

In the first cadaver with successful perirenal spacer placement, these bowel radiation dose reductions were observed: the maximum dose (D_max_) to the small intestine decreased from 25.1 Gy to 22.7 Gy, and the D_2cc_ (the dose to the most irradiated 2 cm³ of the bowel) decreased from 13.2 Gy to 12.7 Gy. Similarly, the D_max_ of the large intestine decreased from 34.5 Gy to 22.1 Gy, and the D_2cc_ decreased from 20.8 Gy to 15.0 Gy.

These dosimetric improvements are summarized in Table 3 and illustrated in Fig. 3, which compares the VMAT dose distributions before and after placement of the hydrogel spacer. Dose-volume histograms comparing the pre- and post-spacer treatment plans are provided in Fig. E1. A pronounced reduction in high-dose exposure was observed in the large intestine, while the target coverage remained generally comparable between the 2 plans (Table 3).

**Table 3:** Dosimetric comparison before and after perirenal spacer placement

Structure	Dosimetric parameter	Before spacer	After spacer	Absolute reduction (Gy)	Relative reduction (%)
Small intestine	D_max_ (Gy)	25.1	22.7	2.4	9.6
	D_2cc_ (Gy)	13.2	12.7	0.5	3.8
Large intestine	D_max_ (Gy)	34.5	22.1	12.4	35.9
	D_2cc_ (Gy)	20.8	15.0	5.8	27.9
Target	CTV D95 (%)	125.7	129.7	-	-
	CTV D99 (%)	123.8	127.6	-	-
	PTV D95 (%)	109.2	111.8	-	-
	PTV D99 (%)	100.0	100.0	-	-

*Abbreviations:* CTV = clinical target volume; D_2cc_ = dose to the most irradiated 2 cm³; D95 = minimum dose received by 95% of the target structure; D99 = minimum dose received by 99% of the target structure; D_max_ = maximum dose; PTV = planning target volume.

**Figure 3:**
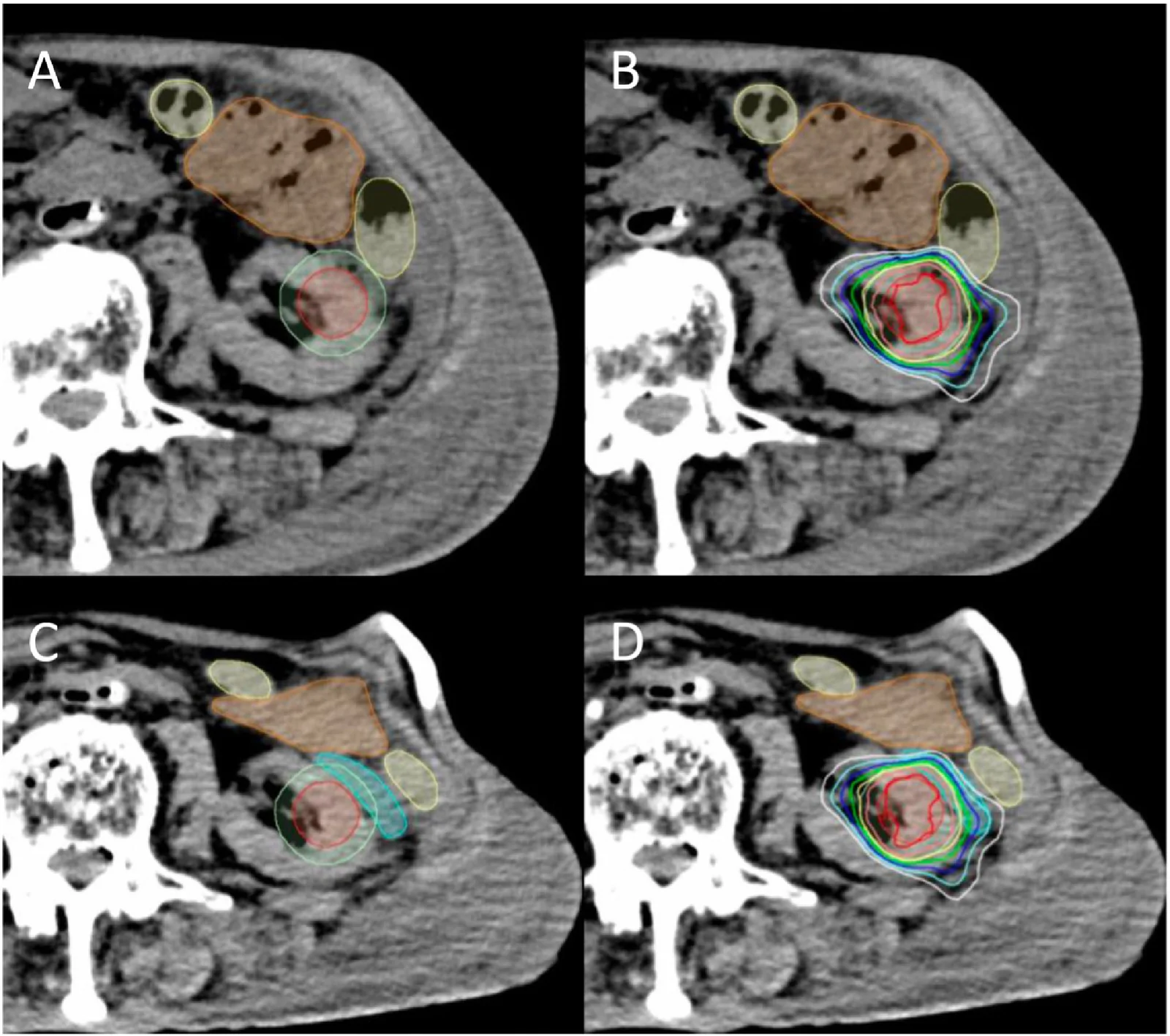
Axial computed tomography (CT) images and volumetric modulated arc therapy (VMAT) dose distributions before and after hydrogel spacer placement. (A) Axial CT image before spacer placement in the perirenal space. (B) Dose distribution from the VMAT plan based on the pre-spacer CT. (C) Axial CT image at 1 month after spacer placement in the perirenal space. (D) Dose distribution from the VMAT plan based on the post-spacer CT at 1 month. The hydrogel spacer, virtual renal cell carcinoma, planning target volume, small intestine, and large intestine are contoured with translucent fills in cyan, red, light green, orange, and yellow, respectively. Isodose lines represent percentages relative to the prescribed dose. High-dose exposure to the bowel structures is reduced in the post-spacer plan (D), while adequate planning target volume coverage is maintained.

## Discussion

Previous studies have demonstrated the potential utility of spacer techniques for facilitating radiation delivery to anatomically challenging abdominal and retroperitoneal regions. Rao et al^14^ demonstrated the feasibility of hydrogel spacer placement between the pancreatic head and duodenum in a human cadaveric model, achieving reproducible separation and improved theoretical dosimetric parameters for SBRT. More recently, Ebrahimi et al^15^ reported successful endoscopic ultrasound-guided placement of a hydrogel spacer to separate an abdominal recurrent cervical cancer lesion from adjacent bowel, facilitating safe SBRT delivery. Yoon et al^16^ reported the clinical feasibility of surgically placed biologic mesh spacers for retroperitoneal and pelvic tumors, enabling displacement of adjacent radiosensitive organs and facilitating external beam radiation therapy. Lee et al^17^ further demonstrated the successful laparoscopic placement of a hyaluronic acid-based spacer in the perirenal space during magnetic resonance-guided SBRT for RCC, resulting in improved target coverage and reduced colonic dose. However, these previous studies primarily focused on achieving separation at specific anatomic sites, and there remains limited information regarding how local anatomic characteristics influence spacer stability and behavior within the retroperitoneum. Therefore, in this preliminary cadaveric study, we compared multiple retroperitoneal sites to explore how the local anatomy may influence hydrogel spacer behavior.

In the perirenal space, the hydrogel remained well-localized and maintained a stable configuration throughout the observation period. This favorable behavior may be attributed to the relatively confined nature of the perirenal compartment, where the fascial boundaries may help maintain the localization of the injected material. These findings are consistent with the clinical report by Lee et al,^17^ who successfully used a perirenal spacer during SBRT for RCC. In contrast, the hydrogel injected into the perivascular region rapidly lost its intended configuration and demonstrated a tendency toward collapse and dispersion. The loose connective tissue and the absence of a well-defined fascial compartment may have permitted redistribution of the injected material along the existing tissue planes. The hydrogel placement adjacent to the iliacus muscle also demonstrated relatively stable retention. However, the clinical relevance of this finding may be limited because this location is less commonly associated with dose-limiting bowel proximity during SBRT. Overall, these observations suggest that local anatomic characteristics may influence spacer performance within the retroperitoneum.

Stable retention of the hydrogel within the perirenal space was associated with favorable dosimetric effects, particularly for bowel sparing during simulated renal SBRT. Although the present study was not designed to assess clinical outcomes or dose-escalation strategies, the observed dosimetric improvements suggest that hydrogel-mediated displacement of adjacent bowel structures may enhance treatment feasibility in anatomically challenging cases and may be relevant when OAR constraints limit treatment planning. Whether spacer-assisted bowel displacement can safely facilitate dose escalation in selected RCC cases remains uncertain and requires further investigation. Given that only a single anteriorly located simulated RCC treated with single-fraction SBRT was evaluated, the magnitude of the dosimetric benefit observed in this study may differ in other clinical situations. Posteriorly located tumors may result in different spatial relationships between the target and adjacent bowel, and therefore, different dosimetric effects. Multifraction SBRT regimens may also yield different dosimetric effects because of differences in dose prescription and OAR constraints compared with single-fraction SBRT. These scenarios warrant further investigation in future studies.

An additional consideration relates to the interpretation of the spacer volume measurements. In the iliacus muscle region, the apparent spacer volume increased at 1 week compared with the immediate postplacement scan. Because hydrogel expansion after polymerization is unlikely, this finding probably does not represent true spacer enlargement. A more plausible explanation is the diffusion of the Gastrografin component into adjacent tissues, resulting in the overestimation of spacer volume on CT imaging. Consequently, the volume retention ratios reported in this study should be interpreted cautiously because the CT-defined spacer volume may not precisely correspond to the actual hydrogel volume. However, the qualitative assessment of spacer morphology and positional stability remained consistent over time, supporting the conclusion that iliacus and perirenal placements maintained their overall anatomic configurations during follow-up.

The present findings require validation in biologically active environments because cadaveric models do not recapitulate the physiological processes that may influence spacer stability and resorption. Future preclinical and prospective clinical studies are needed to determine whether the compartment-specific behavior observed in this study is reproducible in living tissues and associated with clinically meaningful benefits. Such investigations should evaluate the technical feasibility and dosimetric impact. Future clinical studies should carefully evaluate procedure-related complications, including bleeding, infection, injury to adjacent organs, and unintended hydrogel migration, before broader clinical adoption can be considered. Spacer-assisted approaches may also have applications beyond RCC, including selected retroperitoneal malignancies such as sarcomas. Emerging approaches, such as magnetic resonance-guided or adaptive radiation therapy, may also improve treatment feasibility for anatomically challenging RCC. Unlike these technologies, which optimize treatment delivery, spacer placement directly modifies the anatomy and may therefore serve as a complementary strategy for OAR sparing.

This study had some important limitations. First, only 3 cadavers were evaluated, with each retroperitoneal compartment represented by a single specimen. Consequently, reproducibility and interindividual variability could not be assessed, and statistical comparisons could not be performed. Therefore, these findings should be interpreted as preliminary observations rather than definitive evidence regarding the suitability of individual retroperitoneal compartments for spacer placement. Second, although Thiel-embalmed cadavers preserve soft tissue flexibility and anatomic relationships more faithfully than conventional embalming methods, they do not reproduce the key physiological processes present in living patients. Factors such as tissue perfusion, respiratory motion, bowel peristalsis, inflammatory responses, and hydrogel biodegradation may influence the spacer behavior in vivo and alter the stability patterns observed in this study. Third, dosimetric analysis was performed using a single simulated RCC lesion within a cadaveric model. Tumor size, location, adjacent organ anatomy, and fractionation regimen were not systematically evaluated. Therefore, the magnitude of the bowel dose reduction observed in this study may not be generalizable to other clinical scenarios. Given these limitations, the present study should be regarded as an exploratory, hypothesis-generating cadaveric investigation rather than a validation study of clinical feasibility or efficacy. Nevertheless, the comparison of multiple retroperitoneal compartments provides preliminary anatomic insights that may inform subsequent preclinical and clinical investigations.

## Conclusions

This exploratory cadaveric study identified distinct patterns of hydrogel spacer behavior across different retroperitoneal compartments. Stable spacer retention and maintained separation were observed in the perirenal space, whereas spacer collapse occurred in the perivascular region, and anatomically stable but potentially less clinically relevant separation was observed in the iliacus region. In a single simulated RCC SBRT scenario, perirenal spacer placement was associated with reduced bowel dose. These preliminary findings suggest that local anatomic characteristics may influence hydrogel spacer stability within the retroperitoneum and identify the perirenal space as a potentially favorable anatomic environment for further investigation. Additional preclinical and clinical studies are required to determine whether these observations translate into clinically meaningful benefits for living patients.

## Disclosures

Soichi Sugiyama reports financial support (institutional research grant) from PDR Pharma for this work. All other authors declare no competing interests.

## Appendix Supplementary materials

Supplementary material Image, application 1

Figure E1 Image, image 2 dummy alt text
