# Supplementary material for: Preliminary Feasibility and Dosimetric Evaluation of Retroperitoneal Hydrogel Spacer Placement for Stereotactic Body Radiation Therapy: A Cadaveric Study
Source: Adv Radiat Oncol. 2026 Sep 12;12(1):102211. doi: 10.1016/j.adro.2026.102211 (PMC13734874; doi:10.1016/j.adro.2026.102211)
Supplement: Supplementary file 1 [file mmc1.docx]

**Supplementary Material**

**Supplementary Figure E1. Dose–volume histograms comparing treatment plans before and after perirenal hydrogel spacer placement**

The PTV, small intestine, and large intestine are shown in light green, orange, and yellow, respectively. Triangle symbols indicate the pre-spacer plan, whereas square symbols indicate the post-spacer plan.
